# Comparative genomic analysis of six bacteria belonging to the genus *Novosphingobium*: insights into marine adaptation, cell-cell signaling and bioremediation

**DOI:** 10.1186/1471-2164-14-431

**Published:** 2013-06-28

**Authors:** Han Ming Gan, André O Hudson, Ahmad Yamin Abdul Rahman, Kok Gan Chan, Michael A Savka

**Affiliations:** 1 Science Vision SB, Shah Alam, Selangor, Malaysia; 2 The Thomas H. Gosnell School of Life Sciences, Rochester Institute of Technology (RIT), Rochester, New York, USA; 3 Bio Easy SB, Shah Alam, Selangor, Malaysia; 4 Division of Genetics and Molecular Biology, Institute of Biological Sciences, Faculty of Science, University of Malaysia, 50603 Kuala Lumpur, Malaysia; 5 School of Science, Monash University, Sunway Campus, Bandar Sunway, Selangor, Malaysia

**Keywords:** *Novosphingobium*, Marine adaptation genes, Quorum sensing, Aromatic ring hydroxylating dioxygenase

## Abstract

**Background:**

Bacteria belonging to the genus *Novosphingobium* are known to be metabolically versatile and occupy different ecological niches. In the absence of genomic data and/or analysis, knowledge of the bacteria that belong to this genus is currently limited to biochemical characteristics. In this study, we analyzed the whole genome sequencing data of six bacteria in the *Novosphingobium* genus and provide evidence to show the presence of genes that are associated with salt tolerance, cell-cell signaling and aromatic compound biodegradation phenotypes. Additionally, we show the taxonomic relationship between the sequenced bacteria based on phylogenomic analysis, average amino acid identity (AAI) and genomic signatures.

**Results:**

The taxonomic clustering of *Novosphingobium* strains is generally influenced by their isolation source. AAI and genomic signature provide strong support the classification of *Novosphingobium* sp. PP1Y as *Novosphingobium pentaromaticivorans* PP1Y. The identification and subsequent functional annotation of the unique core genome in the marine *Novosphingobium* bacteria show that ectoine synthesis may be the main contributing factor in salt water adaptation. Genes coding for the synthesis and receptor of the cell-cell signaling molecules, of the *N*-acyl-homoserine lactones (AHL) class are identified. Notably, a solo *luxR* homolog was found in strain PP1Y that may have been recently acquired via horizontal gene transfer as evident by the presence of multiple mobile elements upstream of the gene. Additionally, phylogenetic tree analysis and sequence comparison with functionally validated aromatic ring hydroxylating dioxygenases (ARDO) revealed the presence of several ARDOs (oxygenase) in *Novosphingobium* bacteria with the majority of them belonging to the Groups II and III of the enzyme.

**Conclusions:**

The combination of prior knowledge on the distinctive phenotypes of *Novosphingobium* strains and meta-analysis of their whole genomes enables the identification of several genes that are relevant in industrial applications and bioremediation. The results from such targeted but comprehensive comparative genomics analysis have the potential to contribute to the understanding of adaptation, cell-cell communication and bioremediation properties of bacteria belonging to the genus *Novosphingobium.*

## Background

*Novosphingobium* is a genus within the alpha subclass of *Proteobacteria* that was separated from the general genus, *Sphingomonas* a result of extensive classification on the basis of the 16 S rRNA sequence, chemotaxonomic and physiological analyses [1]. Currently, the genus *Sphingomonas* is divided into four genera namely; *Sphingomonas, Sphingobium, Novosphingobium* and *Sphingopyxis*. Bacteria belonging to the genus *Novosphingobium* are often associated with the biodegradation of aromatic compounds such as phenol, aniline, nitrobenzene, 4-chlorobenzene, phenanthrene, pyrene, carbofuran, dibenzofuran and estrogen [2-10]. Given their extensive bioremediation properties these bacteria are frequently isolated from estuarine sediment, coastal sediment and marine aquatic environments that have been exposed to high level of anthropogenic activities.

The recent report of a plant-associated *Novosphingobium* sp. Rr 2–17 isolated from the surface of a crown gall tumor on grapevine has provided an interesting insight regarding the putative role(s) of this genus as a plant epiphyte [11]. Strain Rr 2–17 was found to produce significant amounts of quorum sensing signals of the *N-*acyl-homoserine lactone (AHL) class. A subset of these AHLs has the potential to influence the onset of crown gall disease by pathogen *tumefaciens* in addition to other phytopathogical effects. Recently, *Novosphingobium* sp. AP12 has been isolated from the rhizosphere of *Populus deltoides* as part of the initiatives to understand the metabolic interactions between plants and bacteria belonging to this genus [12].

The common trend in the study of the culturable bacteria belonging to *Novosphingobium* genus typically entails isolation, identification, carbon utilization test and the analysis of extracellular products [3,4]. To a certain extent, mutagenesis experiments has also been employed to elucidate the function(s) of certain genes involved in quorum sensing signal metabolism [11]. Given the well-established phenotypical characterization of various reported *Novosphingobium* strains, it would be of great interest to the scientific community to compare phenotypical characteristics to the genetic make-up of these bacteria. For example, traits associated with xenobiotic compound metabolism, cell-cell signaling and adaptation to marine osmotic condition are very much of interest to further the understanding of the genus pertaining to its lifestyle in certain environments and / or conditions. In addition, the availability of this information has the potential to facilitate targeted gene-specific functional studies in *Novosphingobium* species that are currently unknown.

The present study compares the genomes of six bacteria belonging to the genus *Novosphingobium*. The six bacteria are as follows; *Novosphingobium aromaticivorans* DSM12444 (NC_007794, NC_009426, NC_009427), *Novosphingobium pentaromaticivorans* US6-1 (AGFM010000000), *Novosphingobium nitrogenifigens* Y88 (AEWJ010000000), *Novosphingobium* sp. PP1Y (NC_015579, NC_015582, NC_015583, NC_015580), *Novosphingobium* sp Rr 2–17 (AKFJ010000000) and *Novosphingobium* sp AP12 (AKKE010000000) [12-16]. The diverse isolation source and metabolic property of these strains provides an excellent opportunity to apply comparative genomics to identify the genetic features that differentiate each bacterium within this genus. This study reveals that the quorum sensing system that utilizes the AHL class of signals is not universally present in the genus *Novosphingobium*. In addition, marine adaptation in *Novosphingobium* strains is likely to be based on the organic osmolyte mechanism that is fundamentally different from those reported in common Gram-negative marine bacteria that constantly export Na^+^ ion via the sodium-pumping NADH dehydrogenase Nqr [17,18]. Several dioxygenases putatively associated with the biodegradation of aromatic compounds were identified particularly in the known aromatic compound degrader strains based on both phylogenetic tree and similarity search approaches, providing valuable information for strain engineering in the field of bioremediation. To our knowledge, this is the first comparative genomic analysis of the genus *Novosphingobium* with a focus on the genes associated with quorum sensing metabolism, marine adaptation and bioremediation.

## Results

### General features of the sequenced *Novosphingobium* strains and their correlation with phylogenomic classification

Tables 1 and 2 present the general and genomic features respectively of the strains used in this study. The isolation source of strains PP1Y and US6-1 indicates that both are osmotolerant and this property has been experimentally validated by previous studies [6,7]. In addition, strains PP1Y, US6-1 and DSM 12444 were noted for their extensive ability in the biodegradation of aromatic pollutants. Although both strains Rr 2–17 and AP12 are associated with plants, the difference in their isolation source, epiphytic for strain Rr 2–17 and rhizospheric for strain AP12, may suggest a different role in plant-bacteria interaction and ultimately may lead to significant difference in their genomic features.

**Table 1:** **Strains of the *Novosphingobium *group**

**Strain**	**Isolation source**	**Country**	**Specific features**	**Reference**
Y88	Pulp and paper wastewater	New Zealand	Uptake of Mn and Zn from paper mill effluents and is capable of nitrogen fixation and produces poly-3-hydroxybutyrate	[16]
US6-1	Muddy sediment of a bay	South Korea	Degradation of polycyclic aromatic hydrocarbons, especially benzo(a)pyrene	[6]
Rr 2-17	Crown gall tumour	Hungary	Production of the bacterial signalling molecules of the acyl-homoserine lactone class	[11]
AP12	*Populus deltoides* rhizosphere	United States	Associated with the *Populus deltoides* rhizosphere	[12]
DSM 12444	Subsurface sediments	United States	Ability to grow on toluene, naphthalene and other aromatic compounds	[24]
PP1Y	Surface seawater sample from a closed bay	Italy	Degradation of aromatic hydrocarbons and heterocyclic compounds	[7]

**Table 2:** **Genomic features of the *Novosphingobium *strains**

**Feature**	**Strain within the *Novosphingobium *group**					
	**Y88**	**US6-1**	**Rr 2-17**	**AP12**	**DSM 12444**	**PP1Y**
Chromosome size (bps)	4,148,048	5,344,974	4,539,029	5,611,617	4,233,314	5,313,905
Plasmid size(s) (bps)	n.d	n.d	n.d	n.d	184,462 and 487,268	1,161,602, 192,103 and 48,714
GC%	63.95	63.07	62.71	65.91	65.11	63.26
N50	192,509	117,882	130,074	54,713	n.r	n.r
CDS						
Total	3,801	5,234	4,302	5,214	3,937	4,664
# GO assigned (%)	2,733 (72)	3,330 (64)	2,874 (67)	3,489 (67)	2,805 (71)	3,376 (72)
# EC assigned (%)	989 (26)	1,193 (23)	827 (19)	913 (18)	1,089 (28)	1,339 (29)
# IPR assigned (%)	3,273 (86)	4,155 (79)	3,543 (82)	4,400 (84)	3,435 (87)	4,162 (89)
Calculated median pI of total proteome	6.18	5.86	6.17	5.89	5.98	5.79
tRNAs	49	46	47	45	57	58
# Scaffolds	19	n.r	n.r	n.r	n.r	n.r
# Contigs	77	123	166	187	n.r	n.r

Abbreviations: *GO* Gene ontology, *EC* Enzyme code, *IPR* Interproscan, *CDS* coding domain sequence, *n.r* not relevant, *n.d* not yet determined.

**Figure 1:**
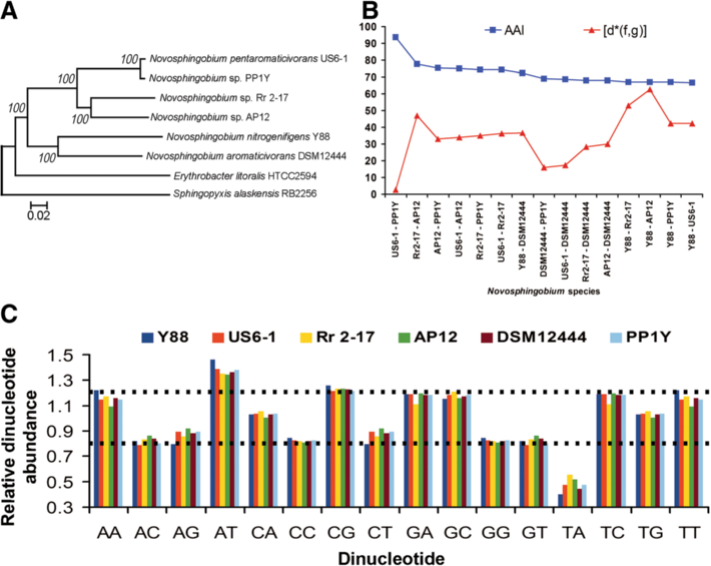
**Genomic taxonomy of *Novosphingobium *strains.** (**A**) Neighborhood joining consensus tree inferred with 1000 replicates constructed using the concatenated MUSCLE alignment adjusted by Gblocks with conservative. The tree is drawn to scale, with branch lengths in the same units as those of the evolutionary distances used to infer the phylogenetic tree. The evolutionary distances were computed using the Poisson correction method and are in the units of the number of amino acid substitutions per site. All ambiguous positions were removed for each sequence pair. There were a total of 310,459 positions in the final dataset. *Sphingopyxis alaskensis* and *Erythrobacter litoralis* were used as outgroups. (**B**) AAI and genome dissimilarity (multiplied by 1000) of different *Novosphingobium* pairs (**C**) Dinucleotide relative abundance in *Novosphingobium* strains. Dotted line indicates the normal range of dinucleotide relative abundance.

The size and G + C content of the genomes used in this study ranged from 4.1 to 5.6 MB and 62.71 to 65.11%, respectively. A slightly lower number and percentage of CDS with gene ontology annotation and assigned enzyme commission numbers were observed for the plant-associated strains Rr 2–17 and AP12 (See Additional file 1 for complete annotation table). The phylogenomic clustering of each strain was of high confidence (100% bootstrap support) and generally correlated well with the isolation source of the *Novosphingobium* strains (Figure 1A and Table 2). However, when a 16S-rRNA based phylogenetic tree was constructed using the same subjects, a slightly different topology with lower bootstrap support was observed instead (Additional file 2A). In addition, a decrease in the overall resolution of the tree was observed when more 16S rRNA sequences of additional *Novosphingobium* species were included into the 16S rRNA based phylogenetic tree analysis (Additional file 2B).

### Average amino acid identity, dinucleotide relative abundance values (ρ*) and genome signature dissimilarity [δ*(f,g)] support phylogenomic classification and provide insights into niche adaptation

The AAI shared among different genome pairs ranged from 66.7% to 93.7% (Figure 1B). Strain PP1Y which is not classified to a species shared a strikingly high AAI (93.7%) with strain US6-1, suggesting that it may belong to the species *Novosphingobium pentaromaticivorans*. Strains Y88 and DSM12444 shared the highest AAI (72%) among themselves as compared to pairing with any other strains ( < 69%) thus providing additional support to the placement of strains Y88 and DSM12444 in a separate cluster in the phylogenomic tree.

The [δ*(f,g)] values within each strain ranged from 3 to 63 (Figure 1B). Strain PP1Y shared the lowest genome dissimilarity value with strain US6-1, again suggesting its identity at the species level as *Novosphingobium pentaromaticivorans*. The calculated ρ* values were in the normal range (0.78 < ρ* < 1.23) for all six strains studied except for AT (over-represented, ρ* > 1.30) and TA (under-represented, ρ* < 0.55). Notably, strain Y88 exhibited the highest ρ*_AA,_ ρ*_TT_ and ρ*_TA_ and the lowest ρ*_AT_ in comparison to other *Novosphingobium* strains (Figure 1C).

### Pan-genome analysis reveals a high abundance of singletons among all strains

**Figure 2:**
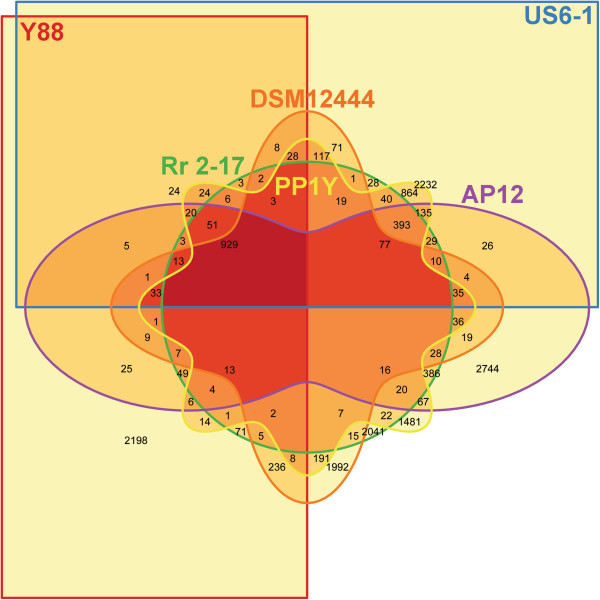
**Pangenome overview.** Six-way Venn diagram showing the number of shared and specific CDSs among the *Novosphingobium* strains. Orthologous grouping were based on 65% identity cut-off and overlapping of at least 70% protein sequence length.

Orthologous clustering for the pan-genome analysis was performed using PanOCT. In comparison to other existing orthologous gene clusters determination tools, PanOCT utilizes a new parameter termed *conservation of gene order* in addition to BLAST score ratio to effectively reassign groups of paralogs into separate clusters of orthologs based on a weighted scoring scheme. At a conservative percentage identity cut-off of 65%, a core genome of strains in the genus *Novosphingobium* containing 929 orthologous groups was identified (Figure 2 and Additional file 3). For strains DSM12444 and PP1Y that have complete genome sequences available, the origin of the singletons was determined. In strain PP1Y, 47% of the singletons are of plasmid origin. However, in strain DSM12444, only 15% of the singletons were located on the plasmid (data not shown). The most closely related marine strains US6-1 and PP1Y shared the largest unique core genes (864 orthologous groups) that will serve as an initial gene pool for the identification of marine adaptation genes in the genus *Novosphingobium*.

### Ectoine biosynthesis genes relevant to salt tolerance are conserved in the marine strains

Two approaches were used to identify the genes that are associated with salt water adaptation in the marine *Novosphingobium* strains (strains PP1Y and US6-1). First, the whole proteome was searched against a BLAST database consisting of sodium-pumping NADH dehydrogenase Nqr (NqrA-F) commonly associated with sodium dependence in Gram-negative marine bacteria and clinical pathogens [18]. However, no significant hit was observed (% identity > 30, E-value < 0.000001). Second, given the identification of the unique core genome of strains PP1Y and US6-1 (Figure 2), CDSs in the unique core genome were subject to SEED annotation that subsequently led to the identification CDSs associated with ectoine biosynthesis in both marine strains (data not shown).

**Figure 3:**
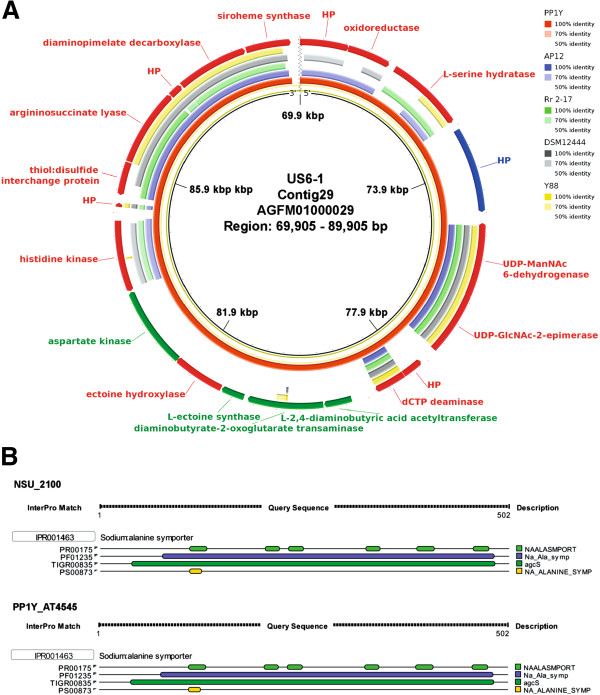
**Marine adaptation genes in the marine *Novosphingobium *strains.** (**A**) Partial genomic map of *Novosphingobium* strains containing the ectoine biosynthesis gene cluster. The inner ring represents the reference sequence, a 20 kb region of marine strain US6-1. Outer shows the shared identity if present according to BLASTN with an E-value cut-off of 0.001. Green arrows indicate genes associated with ectoine biosynthesis. Blue arrow indicates a gene coding for hypothetical protein that may be relevant to salt-tolerance. (**B**) Interproscan protein domain scanning result suggesting the presence of sodium: alanine symporter function in the hypothetical proteins (Blue arrow, **A**) which are uniquely shared by the marine strains US6-1 and PP1Y.

A 20-kbp region of contig29 from strain US6-1 containing the ectoine biosynthesis gene cassette was compared against the whole genome sequences of *Novosphingobium* strains. Genes related to ectoine synthesis (green-labeled arrows, Figure 3A) were conserved only in the marine strains US6-1 and PP1Y (Figure 3A). Ectoine is a type of highly water-soluble organic compound that can accumulate in the cell without significant effects on the cell’s metabolism even at high cytoplasmic concentrations [19]. After the accumulation of ectoine, the diffusion of water through the cell membrane can be reduced thus enabling the survival of cells at high salt environment. The synthesis of ectoine involves three main proteins namely 2,4-diaminobutyric acid (DABA) transaminase (EctB), DABA acetyltransferase (EctA) and ectoine synthase (EctC). EctB transfers an amino group from glutamate to aspartate-semialdehyde to form DABA. Subsequently, an acetyl group is transferred to DABA from EctA. The cyclic condensation of *N*-acetyl-L-2,4-diaminobutyric acid by EctC will lead to the production of ectoine [19]. The EctA homologs, PP1Y_AT4594 and NSU_2105, in strains PP1Y and DSM12444 respectively are 162-residue in length and slightly acidic (calculated pI values of 5.51 and 5.28 respectively). This slightly acidic feature is also exhibited in the remaining protein components implicated in ectoine synthesis (calculated pI value of 5.33 to 5.55) (Additional file 4).

Interestingly, genomic region containing a gene coding for hypothetical protein (blue-labeled arrow, Figure 3A) was only conserved in the marine strains. This gene corresponded to NSU_2100 and PP1Y_AT4545 of strains US6-1 and PP1Y respectively. Protein signature recognition search using Interproscan revealed the presence of a signature motif for the sodium alanine symporter in the hypothetical protein (Figure 3B) that may be responsible for the passage of alanine molecules and sodium ions through the cytoplasmic membrane [20,21].

### *LuxRI* homologs are not universally present in the genus *novosphingobium*

**Figure 4:**
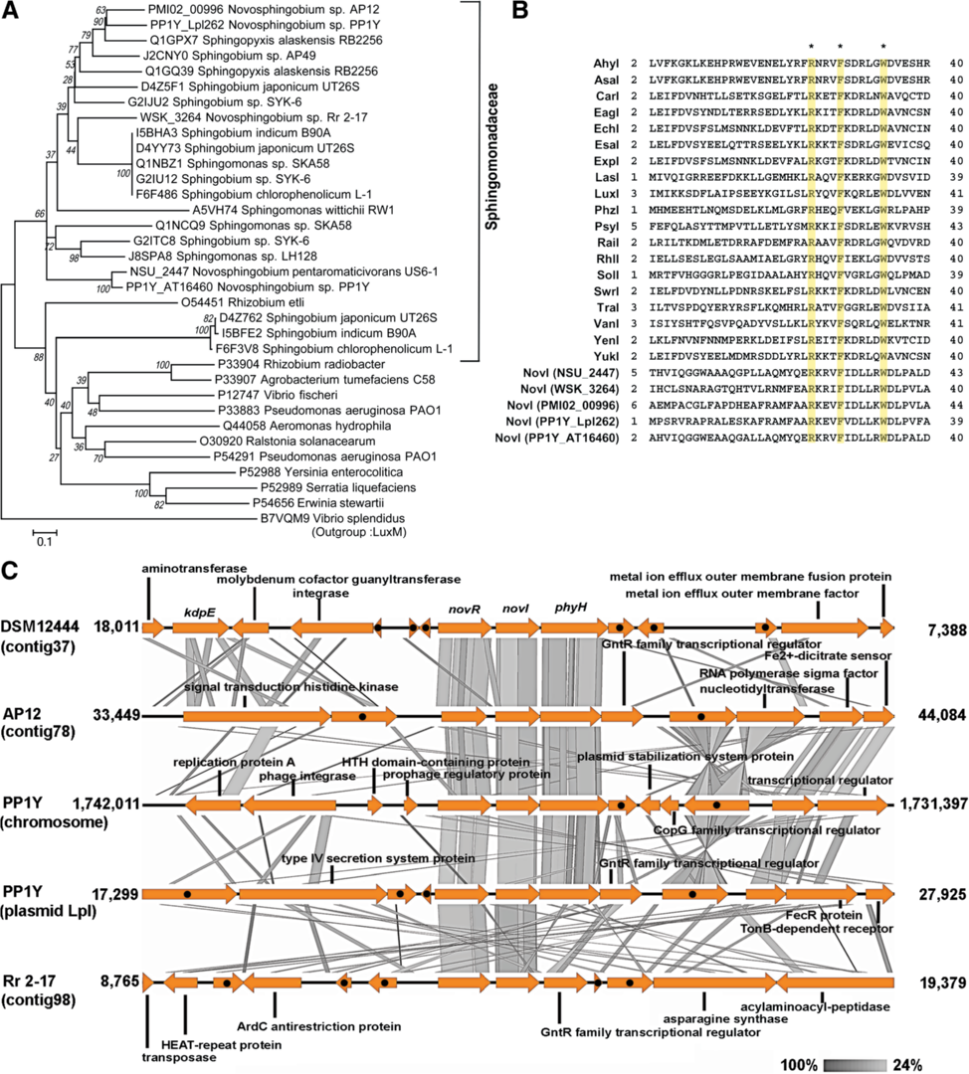
**LuxI homologs in *Novosphingobium *strains.** (**A**) Phylogenetic tree depicting the evolutionary relationship of the curated LuxI homologs and the putative LuxI homologs from *Novosphingobium* strains. (**B**) Alignment of LuxI homologs used in the construction of phylogenetic trees. Asterisk indicates amino acid residues that are 100% conserved in all LuxI homologs. (**C**) Linear comparison of *luxI/R* pairs identified in the *Novosphingobium* strains and the surrounding genes within 5,000 bp of the *luxI* homolog. Arrows with black dots represents genes coding for hypothetical proteins.

In our previous study, *Novosphingobium* sp. Rr 2–17 was found to produce an abundant amount of AHLs that could activate the TraR of *Agrobacterium tumefaciens*[11]. It is therefore of interest to assess the prevalence of genes involved in AHL synthesis in the genus *Novosphingobium*. By performing BLAST searches against the curated LuxI homologs, a total of five putative AHL synthases were identified in strains US6-1 (NSU_2447), PP1Y (Lpl262 and AT16460), Rr 2–17 (WSK_3264) and AP12 (PMI02_00996). Based on phylogenetic genetic tree analysis, the AHL synthases did not exhibit a tight clustering and were dispersed in the monophyletic group consisting of various AHL synthases from the family *Sphingomonadacaea* (Figure 4A). Alignment of the protein sequences with LuxI homologs showed that similar to all curated LuxI homologs, containing the three absolutely conserved amino acids, Arg24, Phe28 and Trp34 (Figure 4B). All identified *luxI* homologs in *Novosphingobium* (*novI)* genes are genetically linked to their cognate transcriptional regulator *novR* that encodes for a LuxR-type transcriptional regulator (Figure 4C). It should be highlighted that *phyH,* that encodes for phytanoyl-CoA dioxygenase, is located adjacent to *novI* in four out of five of the *novI/novR* pairs.

### Identification of a *luxR* solo homolog in *Novosphingobium* sp. PP1Y and evidence for its acquisition through horizontal gene transfer

**Figure 5:**
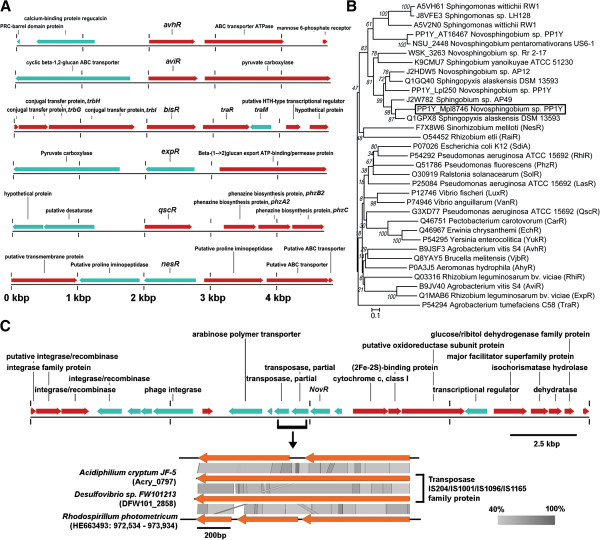
***luxR *solo homolog in *Novosphingobium *sp. PP1Y and its distinctive gene neighborhood.** (**A**) Visualization of the genomic region within 2,000 bp of various well-established *luxR* solo homologs. *avhR*, NC_011989: 3,701,490 - 3,706,230; *aviR*, NC_011989: 774,222 – 778,914; *bisR,* AY177751: 3,858 – 8,598; *expR*, HE995405: 3,197,265 – 3,202,005; *qscR,* NC_002516: 2,067,490 – 2,072,203; *nesR*, NC_020528: 3,032,865 – 3,037,713 (**B**) Phylogenetic clustering of various LuxR homologs identified in the genus *Novosphingobium*. NovR solos were shown in boxed lines. (**C**) 20-kb genomic fragment of strain PP1Y showing the presence of various mobile elements upstream of the *novR* solo and tBLASTx comparison of the presumably fragmented transposase genes immediately upstream of *novR* solo with two complete transposase genes and also a genomic region of *Rhodospirillum photometricum*.

Filtering based on the Interproscan identifier IPR005143 (Transcriptional factor LuxR-like, auto inducer binding domain) in the proteome of all six *Novosphingobium* strains indicated the presence of an additional NovR (PP1Y_Mpl8746) in strain PP1Y which is closely related to a LuxR homolog of *Sphingopyxis alaskensis* RB2256 (UniProt ID: Q1GPX8) (Figure 5B). Analysis of the gene neighborhood of the *novR* strain PP1Y confirmed the absence of *novI* in its vicinity, suggesting its role as a *novR* solo*.* Additionally, a large abundance of mobile elements such as transposases and phage integrases were located upstream of *novR* (Figure 5C) which was not a typical feature of various previously reported *luxR* homolog solo (Figure 5A)*.* Two transposase genes located immediately upstream of *novR* were found to code for different fragment of a transposase, suggesting that the genomic region encompassing both partial transposase genes may code for a functional transposase at one time.

### Identification of various aromatic ring hydroxylating dioxygenase (ARDO) broadens the bioremediation potential of *Novosphingobium* strains

**Figure 6:**
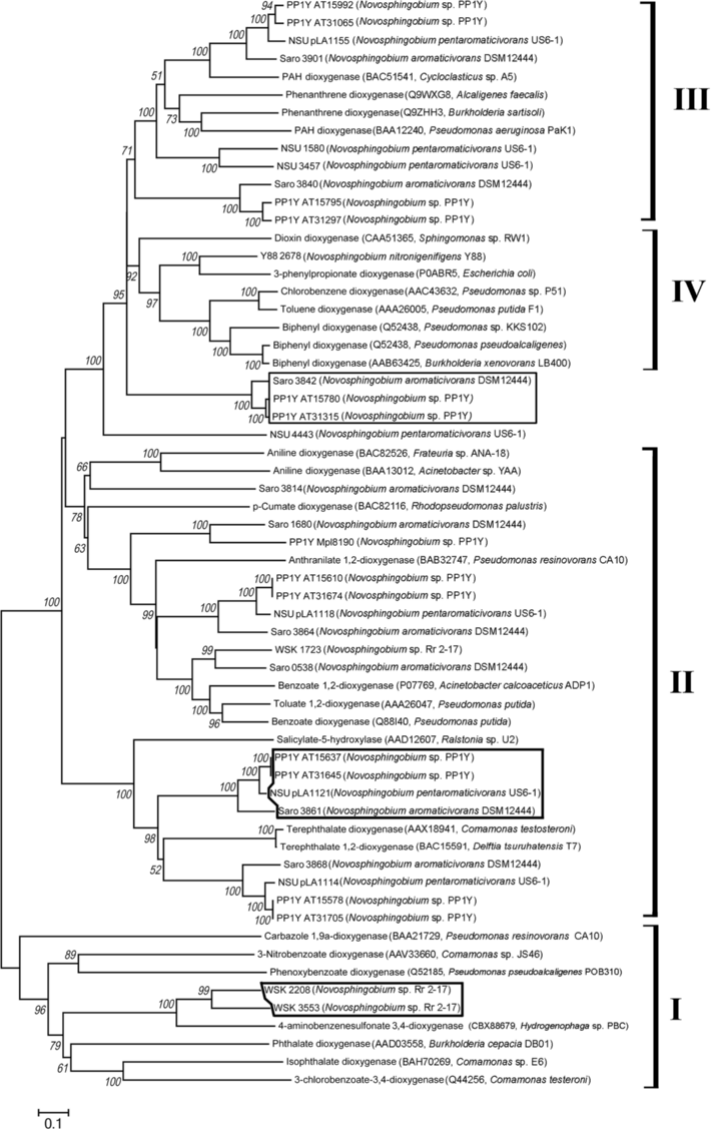
**Neighbor-joining phylogeny inferred from aligned amino acid sequences of ARDOs.** ARDOs forming a monophyletic group with previously validated and clustered ARDOs are considered to be of the same group. Bootstrap values equal to or greater than 50% are shown, and the scale bar represents the number of substitutions per site. For the functionally validated ARDOs, the accession number is shown directly next to the dioxygenase followed by its isolation source. ARDOs shown in boxed lines exhibited distinctive characteristics and are referred to in the text.

ARDO has always been implicated in the biotransormation of aromatic carbon into less toxic compound [22,23]. Through the action of aromatic ring hydroxylation, oxygen is incorporated into the ring structure that further destabilizes the structure and promotes ring cleavage in the ensuing enzymatic step. A phylogenetic tree consisting of functionally validated aromatic ring hydroxylating dioxygenase (ARDO) and the putative ARDOs from *Novosphingobium* strain identified based on similarity search was constructed (Figure 6). As expected, a higher abundance of ARDOs were found in strains US6-1, PP1Y and DSM 12444 that are validated aromatic compound degraders [6,7,24]. Interestingly, Saro_3842 (strain DSM12444), PP1Y_AT15780 and PP1Y_AT31315 (strain PP1Y) formed a separate cluster that shared a common ancestor with Group 3 and Group 4 ARDOs, suggesting potentially a new group of ARDOs (Figure 6, top line box). The alignment of all ARDOs used in this analysis revealed an interesting feature in four ARDOs namely NSU_pLA1121 (strain US6-1), Saro_3861 (strain DSM12444), PP1Y_AT15637 and PP1Y_AT31645 (strain PP1Y). Instead of having 17 amino acids between the first histidine and the second cystiene residues in the Rieske-Type [2Fe-2S] cluster binding site sequence typical of Group II ARDO, there are 19 amino acids separating these residues (Figure 6, middle line box and Additional file 5).

Three ARDOs, two belonging to Group I ARDO, and one belonging to the Group II ARDO, were identified in strain Rr 2–17 that was not known for its biodegradation ability. The identified Group I ARDOs of strain Rr 2–17 which interesting were the only Group I ARDOs of *Novosphingobium* origin in this study formed a monophyletic group with the oxygenase component of 4-aminobenzenesulfonate 3,4-dioxygenase (Figure 6, bottom line box). Similar to the 4-aminobenzenesulfonate 3,4-dioxygenase operon of *Hydrogenophaga* sp. PBC, a gene coding for glutamine synthase that may be involved in the amino group transfer of aromatic amines was located directly next to both of the ARDO genes [25] (data not shown).

## Discussion

The elucidation of evolutionary relationship via phylogenomic approaches provides a very high confidence clustering when compared to the more facile 16S rRNA based approaches. Furthermore, the decrease in the resolution of 16S rRNA based phylogenetic tree upon the inclusion of the more *Novosphingobium* 16S rRNA sequences suggests that single locus-based phylogenetic tree construction may not provide enough information to accurately distinguish evolutionary relationship among member of the *Novosphingobium* genus [26]. AAI and Karlin’s genomic signature dissimilarity generally agreed with the phylogenomic clustering and provided support to the classification of *Novosphingobium* sp. PP1Y as *Novosphingobium pentaromaticivorans* PP1Y. Future whole genome sequencing of additional *Novosphingobium* strains will greatly aid in the determination of the cut-off values necessary to enable a WGS-based species definition as demonstrated by Thompson *et al.*[27]. It is worth noting that strain Y88 which was isolated from a bioreactor treating pulp and paper-mill effluent has the highest AA + TT dinucleotide frequency among the strains studied. In *Pasteurella multocida*, the continuous culturing at higher temperatures led to increased AA, TA, and TT frequencies in comparison with the virulent strain [28]. Since the conformation of AA/TT dinucleotides is not heavily altered by its adjacent nucleotide, these dinucleotides are more stable when exposed to high temperature [29]. The genomic feature exhibited by strain Y88 may therefore correspond to an adaptation to the continuous exposure to mill effluent which is generally above ambient temperatures [30].

Pan-genome analysis based on a conservative percent identity cut-off of 65% revealed a large abundance of singletons in the genus *Novosphingobium* and a rather small core genome (N = 929 genes). It should be noted that the pan-genome analysis was performed using strains of the same genus and not strains of the same species thus explaining the small core genome. A further significant reduction in the core genome and number of singletons in each strain is very likely with the addition of more newly sequenced strains in the future. For example, a core genome of up to 1520 genes has been reported for strains of the species *Vibrio cholerae* and the inclusion of more genomes from different *Vibrio* species (32 strains in total) further reduced the core genome down to 488 genes. Nevertheless, combining the prior knowledge of the wet-lab validated phenotypical trait of the bacterial strains and the identification of unique core genome from such an unorthodox pan-genome analysis, we were able to systematically determine the putative proteins that are involved in marine adaptation. Such approach reinforced the idea that unique core genome should reflect the phenotypical traits that are specific to a group of bacteria [31]. An additional similarity search directly against the nucleotide sequences consisting of the bacterial whole genome sequences eliminated the possible presence of homologous genes that were not included in the orthologous grouping either due to gene prediction failure or partial sequence length.

Due to the lack of active transport system for water in many microorganisms, the biosynthesis of compatible solutes such as ectoine in the marine *Novosphingobium* strains is advantageous in regulating osmotic balance across the cell membrane. Based on molecular dynamic simulations, ectoine has been shown to be a durable water structure-forming solute across a broad temperature range [32]. The effectiveness of ectoine has been demonstrated in the halo tolerant *Brevibacterium* sp. JCM 6894 [33]. Upon osmotic shift towards higher salinity, the supplementation of ectoine has been shown to provide the most remarkable growth stimulation independent of the range of osmotic shifts compared to other compatible solutes,. *Sphingopyxis alaskensis* was the first member in the family of S*phingomonadaceae* reported to possess the complete gene set for synthesis of ectoine.[34]. Subsequently, based on BLASTP query, it appears that at least one member in each of the genus *Sphingomonas*, *Sphingobium* and *Novosphingobium* possesses such gene set (data not shown), suggesting that ectoine-based organic osmolyte mechanism could be a fairly common strategy employed by marine sphingomonads to persist in high-salt environments.

The gene coding for hypothetical protein containing the sodium/alanine symporter protein motif, which is present only in the marine *Novosphingobium* strains US6-1 and PP1Y, may also be involved in osmoregulation. In *Desulfovibrio vulgaris*, the gene coding for sodium/alanine symporter was overexpressed upon exposure to salt shock [35]. In addition, the accumulation of alanine in cells was concurrent with the alleviation of salt-related growth inhibition. Alanine has also been reported to play a role in the relief of salt mediated inhibition on the enzymes involved in nitrogen fixation, photosynthesis and respiration of nitrogen-fixing cyanobacterium [36].The slightly acidic proteome (average calculated median pI of 5.8) and the presence of compatible solute synthesis in both marine sphingomonads closely mimic the salt adaptation strategy employed by *Halomonas elongata*[34] thus supporting the notion that highly acidic proteome (pI < 5.0) [37] is not required for salt adaptation provided that there is a mechanism to accumulate and/or synthesize osmolyte in the cell.

The presence of conserved amino acids hypothesized to be essential for conformational change during substrate binding [38], provides significant bioinformatics support that the five LuxI homologs identified in this study are potentially capable of AHL signal production. It would be important to further genetically and biochemically validate the function of the putative AHL synthase and determine the identity of their AHL signals. The alignment of gene context / neighborhoods of and around the *luxI* homologs show conserved topology where *novR* is proximal to *novI* and where transcriptional orientation is convergent with the receptor (*novR*) upstream (of *novI*), a genomic context feature based on proximity commonly found in complete QS regulatory circuits (*luxR* and *luxI*) in the alpha subclass of Proteobacteria. In addition, this feature is also generally found in the genomes of Proteobacteria [39]. Interestingly, downstream of the *novI* genes in four of the five aligned contents showed a gene coding for phytanoyl-CoA dioxygenase (*phyH*) whose expression is convergent with the QS regulatory circuits. Such topology has also been observed in a fosmid metagenomic library clone and in other genera within *Sphingomonadaceae* including, *Sphingomonas* and *Sphingopyxis*[11]. Based on transposon insertion mutagenesis and the short intergenic distance between *luxI* homolog and *phyH*, it was hypothesized that *phyH* is a gene in an operon that is QS regulated [40]. PhyH belongs to the family of iron(II)-dependent oxygenases and is responsible for the alpha-hydroxylation of phytanoyl-CoA, a derivative of phytanic acid.

The grapevine crown gall tumor epiphyte strain Rr 2–17 possess a single AHL synthase homolog although it has been previously demonstrated that strain Rr 2–17 could produce AHLs of various chain lengths [11]. Three possible reasons could lead to this observation. First, this AHL synthase may contain a rather broad specificity for its acyl-carrier counterpart as previously reported for some AHL synthases [41,42]. Second, additional genes coding for AHL synthases were not identified due to sequence divergence. Third, it is also possible that one or more of the AHL synthase genes were lost in the gaps of the draft genome. Further study entailing targeted disruption of the AHL synthase gene in strain Rr 2–17 and assessment of AHL production in the mutant strain will be necessary to validate these hypotheses. The successful construction of the AHL negative mutant will also be beneficial for future work involving whole transcriptome sequencing since this will directly provide insights into the genes that are regulated by quorum sensing in the genus *Novosphingobium.*

The identification of a putative *novR* solo which was flanked upstream by various mobile elements suggests that its acquisition is via horizontal gene transfer. Two possible mechanisms were proposed for the acquisition of *novR* solo. First, the *novR* solo may be part of the gene cassettes incorporated into an integron as evident by the presence of various genes coding for recombinase upstream of *novR* solo. Second, given the presence of two fragmented transposase genes upstream of *novR* solo, it is also plausible that *novR* solo was acquired via transposition and the subsequent transposase inactivation possibly due to a combination of nonsense and frame shift mutations prevented further movement of the gene. Unlike the *novR* solo, transposase elements were absent in the close vicinity of various well-studied *luxR* homolog solo [43].

The association of mobile element with the complete *luxIR*-type quorum sensing system has been previously reported. For example, *spnIR* has been reported to be localized in a mobile transposon and was found to regulate the transposition frequency [44]. In addition, *luxI* and *luxR* homologues which were separated by transposase genes have also been reported previously in a metagenomic library clone [45]. The solo NovR (PP1Y_Mpl8746) in strain PP1Y could function in eavesdropping on AHL production within microbial communities and / or enable additional AHL regulated gene expression in itself. This identification of a putative LuxR homolog solo in strain PP1Y is similar to BisR of *Rhizobium leguminosarum* and ExpR of *Sinorhizobium meliloti*. It is worth noting that these two bacterial species produce multiple AHL signals and have multiple complete QS regulatory circuits [42,46]. Similarly, two complete QS regulatory circuits, one on the chromosome and one on plasmid, have also been identified in the Italian marine strain, PP1Y [add citation].

The identification of the putative ARDOs and the prediction of their function via phylogenetic analysis correlated particularly well with the remarkable bioremediation capability of strains US6-1, PP1Y and DSM 12444 (Figure 6). High abundance of ARDOs in plasmid pNL2 of strain DSM 12444 is consistent with previous report on the sequencing and analysis of pNL2 [47]. Targeted mutagenesis of the putative ARDOs followed by the assessment of biodegradation ability will be imperative to provide functional analysis data. In addition, assuming that the transcription of ARDO gene sets is tightly regulated as observed in several dioxygenase systems [25,48,49], it is also possible to potentially identify the key ARDO for a specific aromatic compound via differential gene expression analysis. The presence of gene coding for glutamine synthase directly next to the ARDOs of strain Rr 2–17 is particularly intriguing. In the 4-aminobenzenesulfonate 3,4-dioxygenase system, SadB, a glutamine synthase, was hypothesized to catalyze the removal of amine group from the hydroxylated 4-aminobenzenesulfonate to form 4-sulfocatechol based on targeted gene deletion and random transposon mutagenesis studies [25]. Given the fact that 4-aminobenzenesulfonate is a xenobiotic, the identification of the original or natural substrate for this group of ARDOs may be further examined using strain Rr 2–17 that has never been exposed to, to this compound according to the current literature [11].

## Conclusions

The facile sequencing of whole bacterial genomes as a result of next generation sequencing technologies has brought about a paradigm shift in the field of microbiology. The genome analysis of six *Novosphingobium* strains resulted in the identification of various genes putatively associated with the reported phenotype of *Novosphingobium* strains pertaining to salt-tolerance, biosynthesis and perception of cell-cell signaling molecules and aromatic compound biodegradation. Of special mention is the identification of a *luxR* solo that was flanked by various mobile elements, providing new insights into the potential origin of this LuxR solo. The results from this study have the potential to provide information to facilitate future studies relating to the cloning and functional analysis of genes in *Novosphingobium* species.

## Methods

### Genome strains and analyses

The GenBank files containing the genome information of *Novosphingobium* strain were obtained through the NCBI database (http://www.ncbi.nlm.nih.gov) [AEWJ010000000, AGFM010000000, AKFJ010000000, AKKE010000000, NC_007794, NC_009426, NC_009427, NC_015580, NC_015579, NC_015582, NC_015583]. Python script was used to extract the protein sequences for subsequent analysis. The Fasta file of the protein sequences from each genome served as the queries for the BLAST analysis. Visualization of the gene arrangement was done using Gview [50]. The calculation of the protein pI was performed using ProPAS [51].

### Pan-genome analysis

An all-versus-all BLASTP was performed on the extracted protein sequences from each strain. The BLAST output were used as an input for the identification of single-copy orthologs using PanOCT (% Identity 65; E-value < 1e^-10^) [52]. Venerable in R was used to construct the six-way Venn diagram.

### Identification of AHL synthase and aromatic ring hydroxylation dioxygenase

A BLAST database was initially constructed using protein sequences retrieved from UniProt database (Keyword: Acyl-homoserine lactone synthase; Reviewed proteins only). The entire protein sequences from all strains were queried against the database based on E-values < 0.000001. Query hits exhibiting more than 30% identity to any of the AHL synthase were subject to further phylogenetic analysis. To generate a linear comparison of the gene neighborhood around the *luxI* homolog, translated BLAST (tBLASTx) was performed with maximum E-value set to 0.001 followed by map generation with Easyfig 2.1 [53]. For the identification of candidate dioxygenase relevant to bioremediation, a list of functionally validated dioxygenases was used for the construction of BLAST database.

### Identification of marine adaptation genes

From the pan-genome of *Novosphingobium* strains, unique core genes shared between strains PP1Y and US6-1 were extracted and subjected to SEED annotation in MEGAN4 [54]. CDSs associated with osmotic stress were extracted for further analysis. Protein sequences were also searched against a BLAST database that consisted of Nqr-related protein for potential sodium pump. Blast Ring Image Generator (BRIG) was used to visualize the organization of marine adaptation genes in the marine strains and provide support to the absence of the marine adaptation genes in the non-marine strains (% Identity > 50%, E-value < 0.001).

### Phylogenomic and phylogenetic analysis

Hal, an automated pipeline for phylogenomic analysis was used to assess evolutionary relationship based on whole genome protein information [55]. For all phylogenetic analyses, MEGA5 [56] was used instead. Sequences were aligned using MUSCLE [57] and subject to phylogenetic analysis using Neighborhood Joining method [58]. The evolutionary distances were computed using Composite Likelihood method and the Poisson correction method for nucleic acid- and amino acid-based analysis respectively. Positions containing alignment gaps and missing data were eliminated only in pair-wise sequence comparison (pair-wise deletion option). A bootstrap value of 1,000 was used for all phylogeny analyses.

## Competing interests

No competing interests for any of the authors exist.

## Author’s contributions

MAS and KGC supervised this study, contributed to data interpretation and writing of the manuscript. HMG contributed in data gathering, data analysis, data interpretation and writing of the manuscript. AOH contributed to data analysis and writing of the manuscript. AYAR contributed in linear genome comparison. All authors read and approved the final manuscript.

## Supplementary Material

Additional file 1 **Annotated proteome.** Complete list of the predicted proteomes of the strains used in this analysis and their annotation summary. Click here for file

Additional file 2 **16S rRNA based phylogenetic analysis of *Novosphingobium *strains.** (**A**) Evolutionary relationship of the *Novosphingobium* strains used in this study as inferred by Neighborhood-joining method. (**B**) Reduction in the resolution of the 16S rRNA based phylogenetic tree upon inclusion of more publicly available 16S rRNA from members of the similar genus. Branches with less than 50% bootstrap support were collapsed. Click here for file

Additional file 3 **Orthologous group and unique CDS.** Table of orthologous groups and singletons of the strains used in this study at 65% identity cut-off. Click here for file

Additional file 4 **Summary of the proteins involved in ectoine synthesis.** Table containing the protein length, calculated median pI and calculated molecular mass of all four main proteins required for ectoine biosynthesis. Click here for file

Additional file 5 **Alignment of dioxygenases with unexpected separation distance between conserved sites.** Curated dioxygenases containing 16 to 18 amino acids separation between the first conserved histidine residue and the second conserved cysteine residue were aligned with a group of dioxygenases from *Novosphingobium* strains with 19 amino acids separation between the similar conserved sites. Click here for file
