# Supplementary figures and images for: Comparative genomic analysis of six bacteria belonging to the genus Novosphingobium: insights into marine adaptation, cell-cell signaling and bioremediation
Source: BMC Genomics. 2013 Jun 28;14:431. doi: 10.1186/1471-2164-14-431 (PMC3704786; doi:10.1186/1471-2164-14-431)

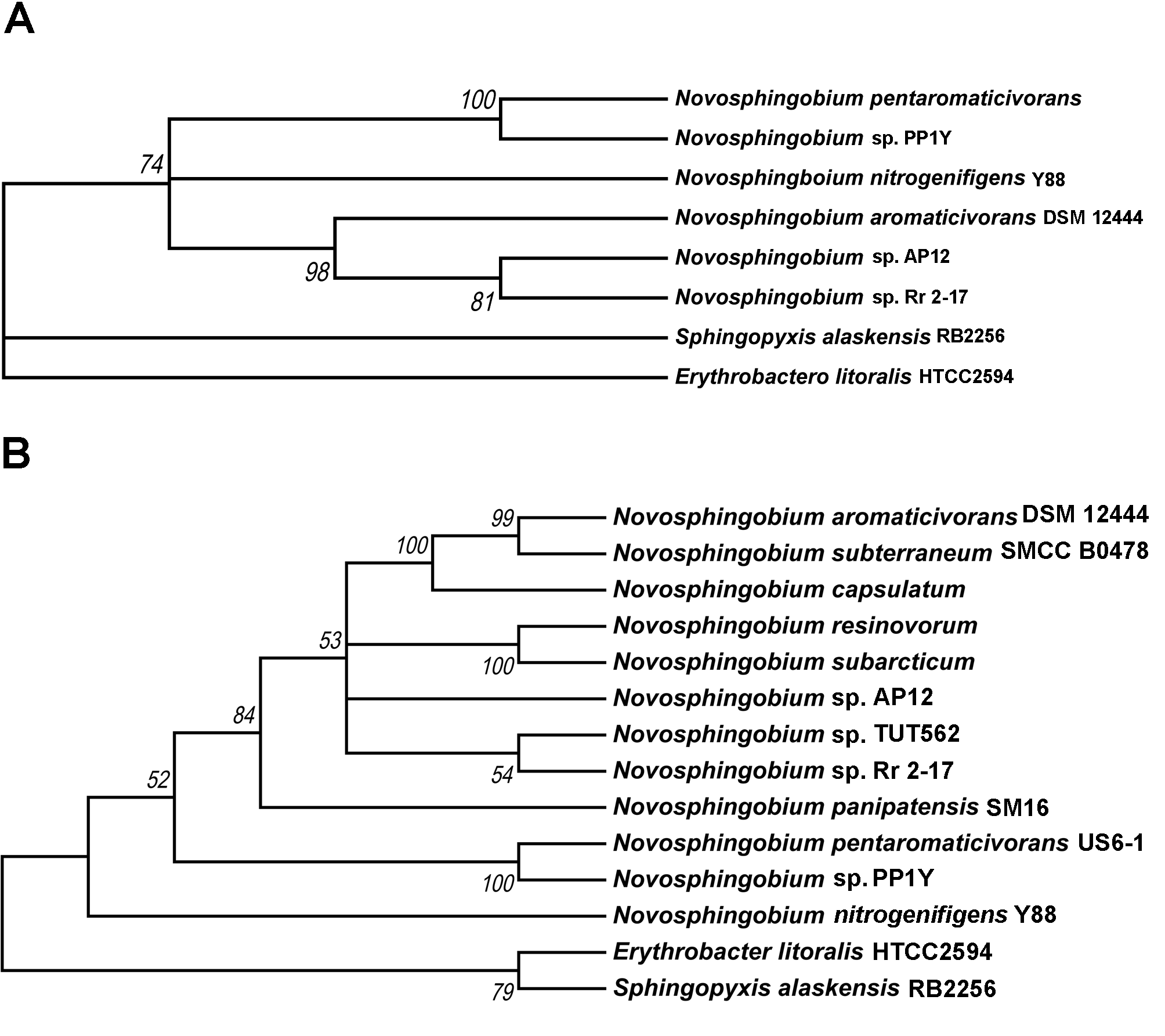

Supplement: Additional file 2 — 16S rRNA based phylogenetic analysis of Novosphingobium strains. (A) Evolutionary relationship of the Novosphingobium strains used in this study as inferred by Neighborhood-joining method. (B) Reduction in the resolution of the 16S rRNA based phylogenetic tree upon inclusion of more publicly available 16S rRNA from members of the similar genus. Branches with less than 50% bootstrap support were collapsed. [file 1471-2164-14-431-S2.tiff]

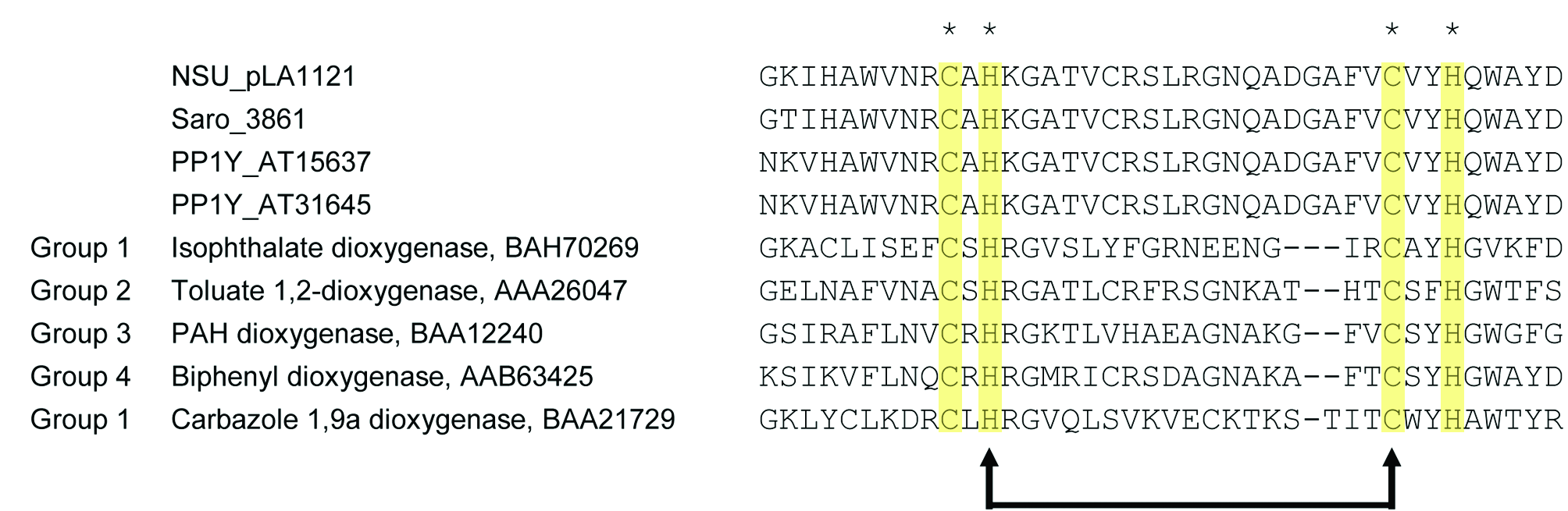

Supplement: Additional file 5 — Alignment of dioxygenases with unexpected separation distance between conserved sites. Curated dioxygenases containing 16 to 18 amino acids separation between the first conserved histidine residue and the second conserved cysteine residue were aligned with a group of dioxygenases from Novosphingobium strains with 19 amino acids separation between the similar conserved sites. [file 1471-2164-14-431-S5.tiff]
