# Supplementary material for: Comparative genomic analysis of six bacteria belonging to the genus Novosphingobium: insights into marine adaptation, cell-cell signaling and bioremediation
Source: BMC Genomics. 2013 Jun 28;14:431. doi: 10.1186/1471-2164-14-431 (PMC3704786; doi:10.1186/1471-2164-14-431)
Supplement: Additional file 4 — Summary of the proteins involved in ectoine synthesis. Table containing the protein length, calculated median pI and calculated molecular mass of all four main proteins required for ectoine biosynthesis. [file 1471-2164-14-431-S4.doc]

| Additional file 1: Property of the proteins involved in ectoine synthesis | | | | |
| --- | --- | --- | --- | --- |
| Protein | Locus tag | Calculated median PI | Calculated molecular mass | Protein length |
| EtcA | pp1y_at4594 | 5.51 | 17522.65 | 162 |
|  | nsu_2105 | 5.28 | 17494.59 | 162 |
| EtcB | pp1y_at4598 | 5.35 | 47652.67 | 442 |
|  | nsu_2106 | 5.35 | 46683.86 | 443 |
| EtcC | pp1y_at4607 | 5.55 | 15294.29 | 137 |
|  | nsu_2107 | 5.4 | 15279.28 | 137 |
| EtcD | pp1y_at4611 | 5.33 | 33936.23 | 304 |
|  | nsu_2108 | 5.49 | 33804.15 | 304 |
